# Neural stem cells and oligodendrocyte progenitor cells compete for remyelination in the corpus callosum

**DOI:** 10.3389/fncel.2023.1114781

**Published:** 2023-01-26

**Authors:** Sarah Moyon, Mara Holloman, James L. Salzer

**Affiliations:** ^1^Department of Neuroscience and Physiology, Institute of Neuroscience, New York University Langone Medical Center, New York, NY, United States; ^2^Department of Neurology, New York University Langone Medical Center, New York, NY, United States

**Keywords:** sub-ventricular zone, neural stem cells, oligodendrocyte progenitor cells, cuprizone, demyelination, remyelination

## Abstract

A major therapeutic goal in demyelinating diseases, such as Multiple Sclerosis, is to improve remyelination, thereby restoring effective axon conduction and preventing neurodegeneration. In the adult central nervous system (CNS), parenchymal oligodendrocyte progenitor cells (pOPCs) and, to a lesser extent, pre-existing oligodendrocytes (OLs) and oligodendrocytes generated from neural stem cells (NSCs) in the sub-ventricular zone (SVZ) are capable of forming new myelin sheaths. Due to their self-renewal capabilities and the ability of their progeny to migrate widely within the CNS, NSCs represent an additional source of remyelinating cells that may be targeted to supplement repair by pOPCs. However, in demyelinating disorders and disease models, the NSC contribution to myelin repair is modest and most evident in regions close to the SVZ. We hypothesized that NSC-derived cells may compete with OPCs to remyelinate the same axons, with pOPCs serving as the primary remyelinating cells due to their widespread distribution within the adult CNS, thereby limiting the contribution of NSC-progeny. Here, we have used a dual reporter, genetic fate mapping strategy, to characterize the contribution of pOPCs and NSC-derived OLs to remyelination after cuprizone-induced demyelination. We confirmed that, while pOPCs are the main remyelinating cells in the corpus callosum, NSC-derived cells are also activated and recruited to demyelinating lesions. Blocking pOPC differentiation genetically, resulted in a significant increase in the recruitment NSC-derived cells into the demyelinated corpus callosum and their differentiation into OLs. These results strongly suggest that pOPCs and NSC-progeny compete to repair white matter lesions. They underscore the potential significance of targeting NSCs to improve repair when the contribution of pOPCs is insufficient to affect full remyelination.

## Introduction

The adult central nervous system (CNS) is subject to sporadic or chronic demyelinating events. Effective remyelination is essential to restore saltatory conduction and to limit neurodegeneration, thereby reversing clinical symptoms following demyelination (Smith et al., 1979; Irvine and Blakemore, 2008; Mei et al., 2016). However, with aging and in demyelinating diseases, such as Multiple Sclerosis, remyelination frequently is insufficient to restore full neurologic function (Shields et al., 2000; Goldschmidt et al., 2009). Accelerating and enhancing myelin repair is therefore a major goal in research to prevent disease progression in such patients.

Remyelination requires activation, expansion and/or differentiation of cells capable of forming new myelin sheaths. In the adult brain, three cell populations have remyelinating capabilities. Parenchymal oligodendrocyte progenitor cells (pOPCs), which are widespread in the adult CNS, are the main pool of remyelinating cells (Zawadzka et al., 2010). Recent studies, in human, primate, murine, and zebrafish models, have also identified mature oligodendrocytes (OLs) as a second pool of remyelinating cells (Yeung et al., 2014; Duncan I. D. et al., 2018; Bacmeister et al., 2020; Neely et al., 2022). However, remyelination by pre-existing OLs tends to be limited compared to newly-formed OLs, at least in murine and zebrafish models (Bacmeister et al., 2020; Neely et al., 2022). Neural stem cells (NSCs) present in the sub-ventricular zone (SVZ) are a third cell population capable of remyelination, as suggested in post-mortem MS tissues and confirmed in animal model diseases (Nait-Oumesmar et al., 1999, 2007; Picard-Riera et al., 2002; Menn et al., 2006; Xing et al., 2014; Brousse et al., 2015; Delgado et al., 2021).

A key question is what determines the contributions of each of these populations to remyelination in demyelinating disease (Franklin et al., 2021). As NSC-derived cells are capable of widespread migration in the CNS (Menn et al., 2006; Xing et al., 2014; Brousse et al., 2015; Butti et al., 2019) and exhibit long-term self-renewal capacities (Ahlenius et al., 2009; Kalamakis et al., 2019; Delgado et al., 2021; Butt et al., 2022), they are interesting source to target for remyelination in aging and in disease. In addition, while pOPC remyelination is characterized by thinner myelin sheaths, remyelination by NSC-derived OLs recapitulates full myelin thickness (Xing et al., 2014; Samanta et al., 2015).

Genetic fate mapping studies indicate that the contribution of NSC-derived cells to remyelination is generally limited compared to pOPCs (Xing et al., 2014; Brousse et al., 2015; Butti et al., 2019). These studies further argue that NSC-derived OLs preferentially remyelinate areas of the corpus callosum (CC) closest to the SVZ (Ortega et al., 2013; Xing et al., 2014; Brousse et al., 2015, 2021; Serwanski et al., 2018; Butti et al., 2019) and do so with a distinct time course (Serwanski et al., 2018). Overall, these studies suggest that pOPCs and NSC-derived cells may compete in time and space with their relative contributions being dictated by the relative abundance, recruitment, and rapidity of repair by the local remyelinating cell populations.

Here, we address directly whether pOPCs and NSC-derived cells compete to repair demyelinated lesions using cuprizone-induced demyelination of the CC as a model. We generated mice that dually report by fate mapping the contributions of pOPCs and NSC-derived cells to remyelination in the CC at different recovery times. We show that both populations are indeed capable of participating in myelin repair, with remyelination carried out preferentially by pOPCs. We also show that the contribution of NSC-progeny to repair is increased when OPC differentiation was genetically blocked. Our results thus show that pOPCs and NSC-derived cells indeed compete to remyelinate the corpus callosum, suggesting that targeting NSCs will be an important strategy to aid repair in aging and in demyelinating diseases when the pOPC remyelination is impaired.

## Materials and methods

### Mouse lines

The animal study was reviewed and approved by the Institutional Animal Care and Use Committee (IACUC) of New York University (NYU) Langone Health. Mice were maintained in a temperature (65–75°F) and humidity (40–60%) controlled facility on a 12-h light–dark cycle with food and water *ad libitum*. On average, equal numbers of mice from each sex were used for this study; we did not note any sex-specificity in our data.

To generate the inducible dual reporter mouse line, we crossed and bred an OPC-specific reporter *NG2-CreER*™** (RRID:IMSR_JAX:008538) (Zhu et al., 2011), *Rosa-CAG-EGFP* (RCE) (RRID:MMRRC_032037-MU) (Sousa et al., 2009) line with the NSC-specific *Nestin-FlpoER*^*T*2^ (MGI:5532191) (Wojcinski et al., 2017), *Ai65 Rosa-CAG-FSF-tdTomato* (FSF-tdT) reporter line (RRID:IMSR_JAX:032864) (Daigle et al., 2018). Heterozygous inducible *NG2*^*CreERTM*/+^;*Nestin*^*FlpoERT*2/+^;*R26*^*RCE*/*FSF–tdT*^ were used as dual reporter mice. For sake of clarity, we abbreviate these mice throughout the manuscript as *NG2-EGFP;Nestin-tdT*.

To generate a conditional inducible knock-out mouse line in which pOPC differentiation is blocked, we crossed and bred the OPC-specific *NG2-CreER*™** driver line (RRID:IMSR_JAX:008538) (Zhu et al., 2011) to the *Myrf-floxed* allele (RRID:IMSR_JAX:010607) (Emery et al., 2009). This line was further combined with the NSC-specific *Nestin-FlpoER*^*T*2^;*Ai65(Rosa-CAG-FSF-tdTomato)* reporter line. Heterozygous *NG2*^*CreERTM*/+^;*Myrf*^*fl*/+^;*Nestin*^*FlpoERT*2/+^;*R26*^*FSF–tdT*/+^ served as controls (abbreviated as *Myrf-Het;Nestin-tdT*) for the homozygous *NG2*^*CreERTM*/+^;*Myrf*^*fl/fl*^;*Nestin*^*FlpoERT*2/+^;*R26*^*FSF–tdT*/+^ conditional knock-outs (abbreviated as *Myrf-cKO;Nestin-tdT*).

### Tamoxifen injections

**FIGURE 1:**
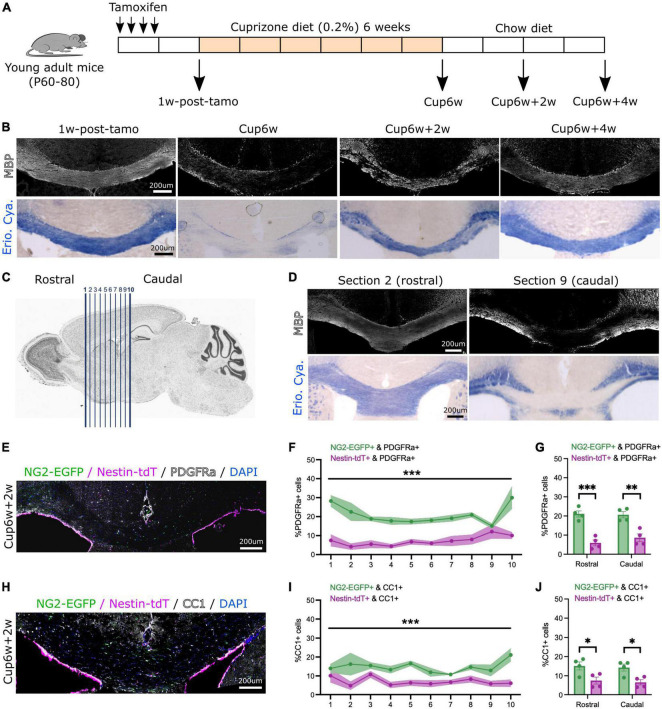
Both neural stem cell-derived oligodendrocytes (OLs) and parenchymal oligodendrocyte progenitor cells (pOPCs) contribute to corpus callosum remyelination. **(A)** Schematic of the experimental design used for our inducible dual reporter *NG2-EGFP;Nestin-tdT* line and inducible conditional knock-out *Myrf-cKO;Nestin-tdT* line. **(B)** Representative images of *NG2-EGFP;Nestin-tdT* coronal brain section immunostained for MBP (in white, top) or stained with Eriochrome Cyanine (Erio. Cya., in blue, bottom), before (1w-post-tamo), after a 6-week cuprizone diet (Cup6w) and after recovery (Cup6w + 2w and Cup6w + 4w). **(C)** Schematic showing serial coronal sectioning of a mouse brain, from rostral (section 1) to caudal (section 10). **(D)** Coronal brain images of section 2 (rostral) and section 9 (caudal) of the same *NG2-EGFP;Nestin-tdT* animal, immunostained for MBP (in white, top) or stained with Eriochrome Cyanine (Erio. Cya., in blue, bottom), after a 6-week cuprizone diet. **(E)** Representative image of *NG2-EGFP;Nestin-tdT* coronal brain section immunostained for PDGFRa (in white) after 2-week recovery (Cup6w + 2w). **(F)** Quantification of the percentages of PDGFRa+ cells that were also NG2-EGFP+ or Nestin-tdT+ for all rostral to caudal CC sections (*n* = 4, lines and shading represent mean ± sem, ***p < 0.001, Two-way ANOVA). **(G)** Average percentages of PDGFRa+ cells that were also NG2-EGFP+ or Nestin-tdT+ for rostral (sections 1–5) and caudal (sections 6–10) sections (*n* = 4, error bars = sem, ***p < 0.001, **p < 0.01, Student’s *t*-test). **(H)** Representative image of *NG2-EGFP;Nestin-tdT* coronal brain section immunostained for CC1 (in white) after 2-week recovery (Cup6w + 2w). **(I)** Quantification of the percentages of CC1+ cells that were also NG2-EGFP+ or Nestin-tdT+ for all rostral to caudal CC sections (*n* = 4, lines and shading represent mean ± sem, ***p < 0.001, Two-way ANOVA). **(J)** Average percentages of CC1+ cells that were also NG2-EGFP+ or Nestin-tdT+ for rostral (sections 1–5) and caudal (sections 6–10) sections (*n* = 4, error bars = sem, *p < 0.05, Student’s *t*-test).

4-Hydroxytamoxifen (Sigma-Aldrich, Burlington, USA, T5648) was dissolved in corn oil (Sigma-Aldrich, Burlington, USA, C8267) at 20 mg/ml for 4 h at 37°C with rotation. To simultaneously activate the *CreER*™* and FlpoER*^*T*2^ recombinases, 250 μL of tamoxifen was administered by intraperitoneal injections to each mouse on days 1, 3, 5, and 7. One week after the last tamoxifen injection (day 14), mice were either euthanized to check recombination efficiency (1w-post-tamo) or started on the cuprizone diet for the experimental groups (illustrated in Figure 1A).

### Cuprizone diet

One week after the last tamoxifen injection (day 14), mice were fed 0.2% cuprizone pellets (Envigo, TD.140800) for 6 weeks. Mice were either euthanized after a 6-week cuprizone treatment (Cup6w) or after additional 2-week (Cup6w + 2w) or 4-week (Cup6w + 4w) recovery periods during which they were fed a normal chow diet (illustrated in Figure 1A).

### Immunohistochemistry

Animals were perfused with 4% paraformaldehyde and post-fixed for 6–12 h in the same solution at 4°C. Brains were dissected, cryo-protected in sucrose solutions, and frozen embedded in OCT. Immunofluorescence was performed on 20 μm cryostat sections. Each slide contained 10 serial sections, from rostral (formation of the corpus callosum) to caudal (level of the fornix) (illustrated in Figure 1C). Slides were washed in PBS 1X, treated with 100% methanol at −20°C for 10 min, then incubated in blocking buffer (10% normal goat or donkey serum in PBS 1X/1% BSA/0.25% Triton-X100) for 1 h at room temperature. Slices were incubated overnight at 4°C with primary antibodies diluted in PBS, 1% BSA, and 0.25% Triton-X100. After rinsing with PBS, sections were incubated with Alexa Fluor secondary antibodies and then washed with PBS. Sections were incubated with Hoechst stain (1:5,000, Thermo Fisher, Waltham, USA, H3569) for 5 min at room temperature. After PBS washes and a final wash in distilled water, stained tissues were cover-slipped in Fluoromount G mounting medium (Southern Biotech, Birmingham, USA, 0100-01).

Primary antibodies used for immunohistochemistry were rat anti-PDGFRα (CD140a, BD, 558774, 1:200) and anti-RFP (Chromotek, Planegg, Germany, 5f8-100, 1:1,000) antibodies; mouse anti-CC1 (APC, Calbiochem, OP80, 1:200) and anti-GFAP (Sigma, Burlington, USA, G3893, 1:400) antibodies; chicken anti-MBP (Millipore, Burlington, USA, AB9348, 1:200) and anti-Nestin (Novus Biologicals, Centennial, USA, NB100-1604, 1:200) antibodies; rabbit anti-Iba1 (Wako, 019-19741, 1:400), anti-EGFP (Invitrogen, Waltham, USA, A11122, 1:500) and anti-NG2 antibodies (Millipore, Burlington, USA, MAB5320, 1:200).

Secondary antibodies used for immunohistochemistry were goat anti-mouse (IgG 680, Jackson ImmunoResearch, West Grove, USA, 115-625-166, 1:1,000), anti-rat (IgG 488, Jackson ImmunoResearch, West Grove, USA, 112-545-167, 1:1,000), anti-rat (IgG 594, Jackson ImmunoResearch, West Grove, USA, 112-585-167, 1:1,000), anti-rat (IgG 647, Jackson ImmunoResearch, West Grove, USA, 112-605-167, 1:1,000), and anti-rabbit (IgG Oregon Green 488, Thermo Fisher, Waltham, USA, O-11038, 1:1,000) antibodies; donkey anti-mouse (IgG 488, Jackson ImmunoResearch, West Grove, USA, 715-546-150, 1:1,000), anti-rat (IgG 594, Jackson ImmunoResearch, West Grove, USA, 712-585-153, 1:1,000), anti-rat (IgG 680, Jackson ImmunoResearch, West Grove, USA, 712-625-150, 1:1,000), anti-rabbit (IgG 488, Jackson ImmunoResearch, West Grove, USA, 711-546-152, 1:1,000), anti-rabbit (IgG 647, Jackson ImmunoResearch, West Grove, USA, 711-605-152, 1:1,000), and anti-chicken (IgY 647, Jackson ImmunoResearch, West Grove, USA, 703-605-155, 1:1,000) antibodies.

Additional myelin staining with Eriochrome Cyanine was performed on 20 μm cryostat sections (Melero-Jerez et al., 2020). Slides were incubated in fresh acetone for 5 min at room temperature and air-dried for 15 min. Slides were stained with 0.2% Eriochrome Cyanine for 25 min, rinsed under running water, then differentiated with 5% ferric ammonium sulfate for 15 min, followed by borax-ferricyanide solution for 10 min. The stained sections were dehydrated in consecutive baths of 70%, 95%, 100% ethanol, and 100% xylene, then cover-slipped in Fluoromount G mounting medium (Southern Biotech, Birmingham, USA, 0100-01).

### Imaging and analysis

Fluorescent slides were examined and acquired on Confocal Zeiss LSM 800 Fluorescence Microscope, using Zeiss Zen blue software. Images were analyzed with Fiji-Image J (RRID:SCR_003070), to quantify numbers of cells, density of cells, and intensity of the staining. All quantifications were performed blindly. Statistical analysis was performed on GraphPad Prism (GraphPad Software, Inc., Boston, USA, RRID:SCR_002798). One-way and two-way ANOVA was used to compare three or more sets of data. For each experiment, *n* = 4–7 biological replicates were quantified (details can be found in each figure caption).

Eriochrome Cyanine stained sections were examined and acquired on Keyence BZ-X710 microscope, using Keyence BZ-Y software.

## Results

### Both neural stem cell-derived oligodendrocytes and parenchymal oligodendrocyte progenitor cells contribute to corpus callosum remyelination

We first addressed the respective contributions of pOPCs and NSC-derived OLs to remyelination of the corpus callosum. We generated a tamoxifen-inducible, dual reporter mouse line by crossing the OPC-specific *NG2-CreER*™** line (Zhu et al., 2011) combined with *Rosa-CAG-EGFP* (RCE) reporter line (Sousa et al., 2009) with the NSC-specific *Nestin*^*FlpoERT2*^ line (MGI:5532191) (Wojcinski et al., 2017) combined with *Ai65 (Rosa-CAG-FSF-tdTomato)* reporter line (Daigle et al., 2018). Post-natal day 60–80 (P60–80) heterozygous inducible *NG2*^*CreERTM*/+^;*Nestin*^*FlpoERT*2/+^*Rosa*^*EGFP/FSF–tdT*^ (named *NG2-EGFP;Nestin-tdT*) received a 1-week course of tamoxifen by intraperitoneal injection, before being started 1 week later on a demyelinating cuprizone diet for 6 weeks (Skripuletz et al., 2008; Figure 1A). Immunofluorescence of the *NG2-EGFP;Nestin-tdT* brain sections (Supplementary Figures 1A–C) 1 week after tamoxifen induction demonstrated that 86.4 ± 1.8% of Nestin+ cells were tdT+ (Supplementary Figure 1B) and 29.7 ± 3.6% of NG2+ cells were EGFP+ (Supplementary Figure 1C). There was no reporter expression in mice injected with corn oil only (Supplementary Figures 1B, C).

We examined in the CC the extent of demyelination induced by the cuprizone diet by immunostaining for MBP or staining with Eriochrome Cyanine (Erio. Cya.) (Figure 1B). Prior to cuprizone treatment (1w-post-tamo), the corpus callosum was strongly myelinated, showing uniform MBP+ and Eriochrome Cyanine staining. In contrast, all animals terminated after 6-weeks of cuprizone (Cup6w) showed extensive demyelination. Myelin in the CC was partially restored 2 weeks (Cup6w + 2w) and 4 weeks (Cup6w + 4w) after switching back to regular mouse chow (Figure 1B, section 5 for each time point). If we did not detect any inter-individual variability from cuprizone, we noticed intra-individual variability, as reported previously (Wu et al., 2008; Xie et al., 2010; Tagge et al., 2016; Zhang et al., 2019). We performed serial sections (from rostral to caudal) (Figure 1C) which demonstrated the extent of demyelination was typically more pronounced in caudal sections (e.g., section 9) compared to rostral sections based on either MBP or Eriochrome Cyanine staining (e.g., section 2, Figure 1D).

Previous studies suggested the peak of pOPC and NSC fate mapped cells in the remyelinating CC is around 2 weeks of recovery (Brousse et al., 2015). Accordingly, we quantified the contributions of NG2-EGFP+ pOPCs and Nestin-tdT+ NSC-progeny to repair at Cup6w + 2w (Figures 1E–H and Supplementary Figures 1D–G). We first quantified the proportion of PDGFRα+ OPCs that were derived from NG2-EGFP+ pOPCs vs. Nestin-tdT+ NSCs for caudal to rostral sections (Figures 1E, F). As expected, for all sections, NG2-EGFP+ pOPCs contribute more to PDGFRα+ cells than do Nestin-tdT+ NSCs (Figure 1F; on average 20.1 ± 3.4% vs. 7.2 ± 2.2%, respectively, and Supplementary Figure 1D; on average 163 ± 41 cells/mm^2^ vs. 59 ± 20 cells/mm^2^, respectively). The difference in contribution was similar in rostral and caudal sections (Figure 1G and Supplementary Figure 1E). We then quantified the proportion of differentiated CC1+ OLs deriving from NG2-EGFP+ pOPCs and Nestin-tdT+ NSCs in caudal vs. rostral sections in the corpus callosum (Figures 1H, I). Again, for all sections, NG2-EGFP+ pOPCs contribute more to CC1+ cells than do Nestin-tdT+ NSC-derived cells (14.7 ± 2.8% vs. 7.0 ± 1.9%, respectively, Figure 1I; 120 ± 29 cells/mm^2^ vs. 59 ± 18 cells/mm^2^, respectively, Supplementary Figure 1F). The difference in contribution was similar in rostral and caudal sections (Figure 1J and Supplementary Figure 1G). These results generally agree with the percentages and densities of pOPCs and NSC-derived cells that contribute to CC1+ OLs at Cup5w + 2w reported in a prior study that used two distinct lines to separately fate map each population (Brousse et al., 2015).

Overall, our results confirmed that pOPCs and, to a lesser extent, Nestin+ NSC-derived cells contribute to remyelination of the corpus callosum following cuprizone-induced demyelination. Because of the relatively low recombination efficiency for the NG2-EGFP reporter, our data likely under-estimates the actual pOPCs contribution. Nevertheless, these results validate the dual labeling strategy and the experimental approach to address potential competition between pOPC population and NSC-progeny during remyelination.

### Cuprizone diet induce similar demyelination of the corpus callosum when parenchymal oligodendrocyte progenitor cells differentiation is blocked

To address whether pOPCs and NSC-derived cells compete during repair, we conditionally knocked-out *Myrf* in pOPCs by crossing the *NG2-CreER*™** driver line with the *Myrf-floxed* allele (Emery et al., 2009; Zhu et al., 2011). Ablation of *Myrf* in pOPCs is known to efficiently block their differentiation (Koenning et al., 2012; Duncan G. J. et al., 2018). We further crossed these alleles to the *Nestin-tdT* reporter line to follow the contribution of NSC-derived cells to remyelination after cuprizone treatment (Figure 1A) in either the cKO mice (*Myrf-cKO;Nestin-tdT*) or in Het controls (*Myrf-Het;Nestin-tdT*). We hypothesized that, if pOPCs and NSC-progeny are competing for repair, blocking pOPCs differentiation should increase the contribution of NSC-derived cells to newly-formed OLs and remyelination.

**FIGURE 2:**
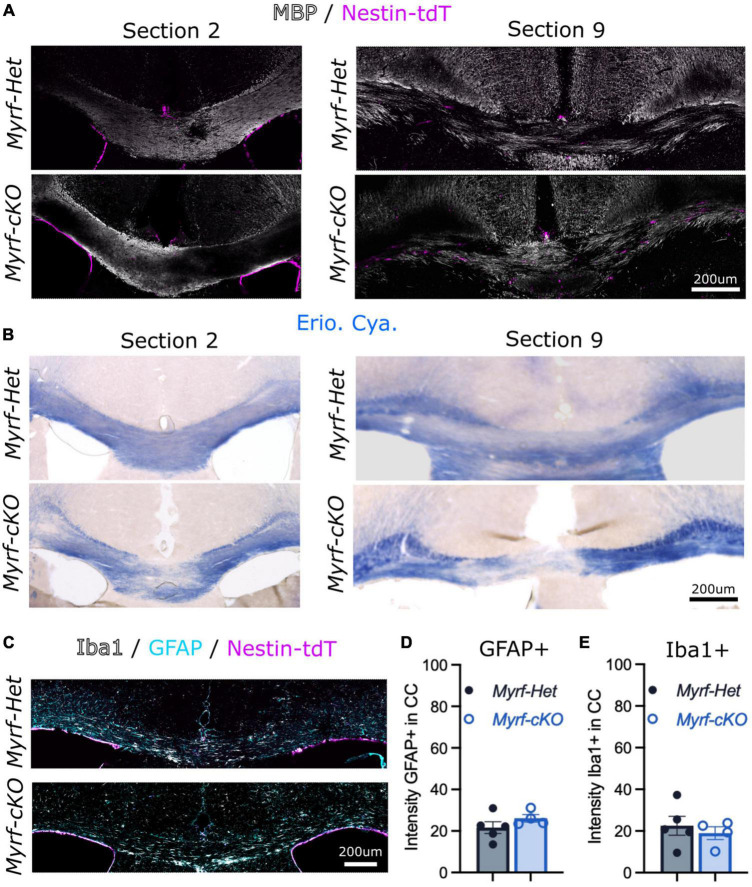
Cuprizone diet induce similar demyelination of the corpus callosum when parenchymal oligodendrocyte progenitor cells (pOPCs) differentiation is blocked. **(A,B)** Representative images of *Myrf-Het;Nestin-tdT* and *Myrf-cKO;Nestin-tdT* coronal brain sections **(A)** immunostained for MBP (in white) and Nestin-tdT (magenta) or **(B)** stained with Eriochrome Cyanine (Erio. Cya., in blue) after a 6-week cuprizone diet (sections 2 and sections 9 of the same animals). **(C)** Representative images of *Myrf-Het;Nestin-tdT* and *Myrf-cKO;Nestin-tdT* coronal brain sections immunostained for Iba1 (in white), GFAP (in cyan) and Nestin-tdT (in magenta) after a 6-week cuprizone diet. **(D)** Quantification of the GFPA+ intensity in the CC of *Myrf-Het;Nestin-tdT* and *Myrf-cKO;Nestin-tdT* brain sections (*n* = 4–5, error bars = sem, Student’s *t*-test). **(E)** Quantification of the Iba1+ intensity in the corpus callosum of *Myrf-Het;Nestin-tdT* and *Myrf-cKO;Nestin-tdT* brain sections (*n* = 4–5, error bars = sem, Student’s *t*-test).

We first confirmed that cuprizone induced similar levels of demyelination and gliosis in the *Myrf-Het;Nestin-tdT* and the *Myrf-cKO;Nestin-tdT* mice based on MBP and Eriochrome Cyanine stainings, and on GFAP and Iba1 stainings (Figure 2). As previously observed, demyelination was more extensive in caudal sections (e.g., section 9) compared to rostral sections (e.g., section 2), in both control and knock-out (Figures 2A, B). Astroglial and microglial activation in the demyelinating region was also comparable in *Myrf-Het;Nestin-tdT* vs. *Myrf-cKO;Nestin-tdT* mice as evidenced by GFAP+ and Iba1+ immunostaining (Figure 2C) and intensity quantification (Figures 2D, E, respectively). These results support using *Myrf-cKO;Nestin-tdT* mice to address NSC-derived cells contribution to remyelination when pOPC differentiation is blocked.

### The contribution of neural stem cell-derived oligodendrocytes to remyelination increases when parenchymal oligodendrocyte progenitor cell differentiation is blocked

**FIGURE 3:**
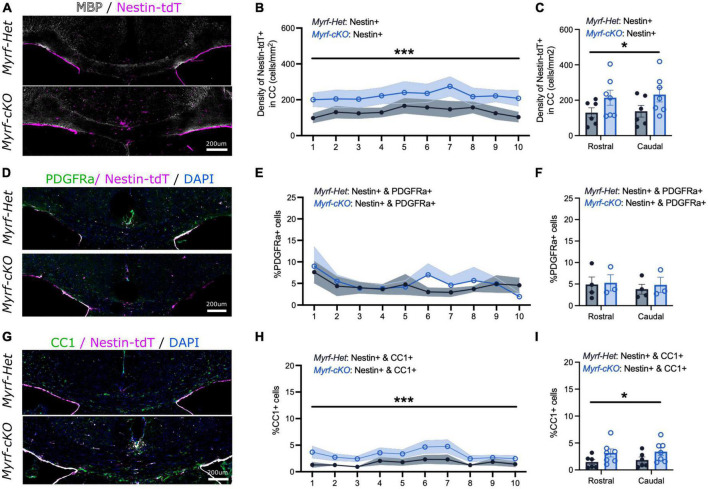
The contribution of neural stem cell-derived oligodendrocytes (OLs) to remyelination increases at 2 weeks of recovery when parenchymal oligodendrocyte progenitor cell differentiation is blocked. **(A)** Representative images of *Myrf-Het;Nestin-tdT* and *Myrf-cKO;Nestin-tdT* coronal brain sections immunostained for MBP (in white) and Nestin-tdT (magenta) after 2-week recovery (Cup6w + 2w). **(B)** Quantification of the densities of Nestin-tdT+ cells within the CC of *Myrf-Het;Nestin-tdT* and *Myrf-cKO;Nestin-tdT* coronal sections (*n* = 6–7, lines and shading represent mean ± sem, ***p < 0.001, Two-way ANOVA). **(C)** Average densities of Nestin-tdT+ cells within the CC of *Myrf-Het;Nestin-tdT* and *Myrf-cKO;Nestin-tdT* rostral (sections 1–5) and caudal (sections 6–10) sections (*n* = 6–7, error bars = sem, *p < 0.05, Two-way ANOVA). **(D)** Representative images of *Myrf-Het;Nestin-tdT* and *Myrf-cKO;Nestin-tdT* coronal brain sections immunostained for PDGFRa (in green) and Nestin-tdT (magenta) after 2-week recovery (Cup6w + 2w). **(E)** Quantification of the percentages of PDGFRa+ cells that were also Nestin-tdT+ in *Myrf-Het;Nestin-tdT* and *Myrf-cKO;Nestin-tdT* coronal sections (*n* = 3–4, lines and shading represent mean ± sem, Two-way ANOVA). **(F)** Average percentages of PDGFRa+ cells that were also Nestin-tdT+ in *Myrf-Het;Nestin-tdT* and *Myrf-cKO;Nestin-tdT* rostral (sections 1–5) and caudal (sections 6–10) sections (*n* = 3–4, error bars = sem, Two-way ANOVA). **(G)** Representative images of *Myrf-Het;Nestin-tdT* and *Myrf-cKO;Nestin-tdT* coronal brain sections immunostained for CC1 (in green) and Nes-TdT (magenta) after 2-week recovery (Cup6w + 2w). **(H)** Quantification of the percentages of CC1+ cells that were also Nestin-tdT+ in *Myrf-Het;Nestin-tdT* and *Myrf-cKO;Nestin-tdT* coronal sections (*n* = 6–7, lines and shading represent mean ± sem, ***p < 0.001, Two-way ANOVA). **(I)** Average percentages of CC1+ cells that were also Nestin-tdT+ in *Myrf-Het;Nestin-tdT* and *Myrf-cKO;Nestin-tdT* rostral (sections 1–5) and caudal (sections 6–10) sections (*n* = 6–7, error bars = sem, *p < 0.05, Two-way ANOVA).

We next quantified the NSC-progeny contribution to remyelination when pOPC differentiation is blocked at an early recovery time point (Cup6w + 2w) (Figure 3 and Supplementary Figure 2). Patchy MBP and Eriochrome Cyanine stainings at Cup6w + 2w suggested the extent of demyelination and initial remyelination was comparable in the *Myrf-Het;Nestin-tdT* and *Myrf-cKO;Nestin-tdT* mice (Figure 3A and Supplementary Figure 2A). We also confirmed that the astroglial and microglial reactivation was similar in both controls and knock-out mice at 2 weeks of recovery (Supplementary Figures 2F, G). Quantification of the number of Nestin-tdT+ fate mapped cells in the CC showed a 1.7-fold increased recruitment in *Myrf-cKO;Nestin-tdT* mice vs. controls (223 ± 40 cells/mm^2^ vs. 134 ± 27 cells/mm^2^, respectively, Figures 3B, C). The increase in the density of NSC-derived cells was similar between rostral and caudal sections, suggesting that recruitment of NSC-progeny could be explained by a general activation of the SVZ area (Figure 3C).

We further assessed whether these newly recruited Nestin-tdT+ NSC-derived cells differentiated into immature PDGFRa+ OPCs (Figures 3D–F and Supplementary Figures 2B, C) or mature CC1+ OLs (Figures 3G–I and Supplementary Figures 2D, E). At Cup6w-2w, the percentages of Nestin-tdT+ cells that were PDGFRa+ were similar for *Myrf-Het;Nestin-tdT* and *Myrf-cKO;Nestin-tdT* CC sections (4.3 ± 1.6% and 5.0 ± 1.8%, respectively, Figure 3E; 39 ± 14 cells/mm^2^ and 36 ± 10 cells/mm^2^, respectively, Supplementary Figure 2B). However, in all sections there was a consistent twofold increase in the contribution of Nestin-tdT+ that became CC1+ OLs in the CC of *Myrf-cKO;Nestin-tdT* compared to controls (3.3 ± 0.7% vs. 1.6 ± 0.5%, respectively, Figure 3H; 50 ± 11 cells/mm^2^ vs. 26 ± 7 cells/mm^2^, respectively, Supplementary Figure 2D). Thus, following block of pOPC differentiation, NSC-derived cells recruitment to and differentiation into OLs in sites of demyelination was enhanced, strongly suggesting that pOPCs and NSC-derived OLs compete to remyelinate lesions. The increased contribution of Nestin-tdT+ NSC-derived cells to OLs (i.e., CC1+ cells) but not pOPCs (i.e., PDGFRa+ cells) at 2 weeks suggest the recruited NSC-derived cells rapidly differentiate into mature and remyelinating OLs rather than simply replenishing pOPCs in the callosum or arresting at the OPC stage.

**FIGURE 4:**
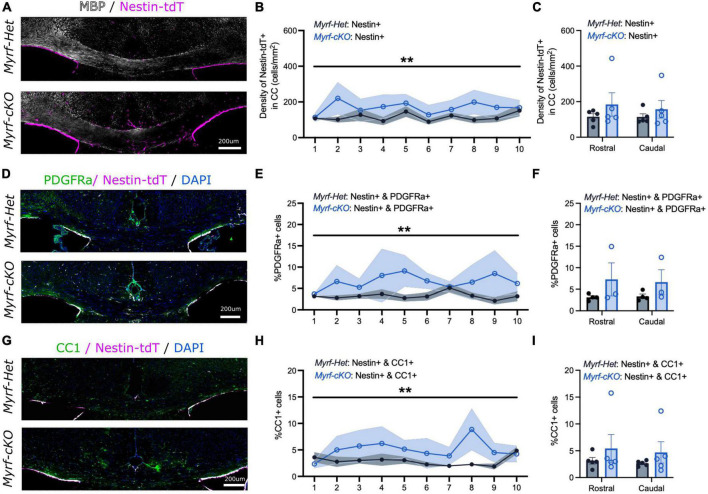
The contribution of neural stem cell-derived oligodendrocytes (OLs) to remyelination remains elevated at 4 weeks of recovery when parenchymal oligodendrocyte progenitor cell differentiation is blocked. **(A)** Representative images of *Myrf-Het;Nestin-tdT* and *Myrf-cKO;Nestin-tdT* coronal brain sections immunostained for MBP (in white) and Nestin-tdT (magenta) after 4-week recovery (Cup6w + 4w). **(B)** Quantification of the densities of Nestin-tdT+ cells within the CC of *Myrf-Het;Nestin-tdT* and *Myrf-cKO;Nestin-tdT* coronal sections (*n* = 5, lines and shading represent mean ± sem, **p < 0.01, Two-way ANOVA). **(C)** Average densities of Nestin-tdT+ cells within the CC of *Myrf-Het;Nestin-tdT* and *Myrf-cKO;Nestin-tdT* rostral (sections 1–5) and caudal (sections 6–10) sections (*n* = 5, error bars = sem, Two-way ANOVA). **(D)** Representative images of *Myrf-Het;Nestin-tdT* and *Myrf-cKO;Nestin-tdT* coronal brain sections immunostained for PDGFRa (in green) and Nestin-tdT (magenta) after 4-week recovery (Cup6w + 4w). **(E)** Quantification of the percentages of PDGFRa+ cells that were also Nestin-tdT+ in *Myrf-Het;Nestin-tdT* and *Myrf-cKO;Nestin-tdT* coronal sections (*n* = 3–4, lines and shading represent mean ± sem, **p < 0.01, Two-way ANOVA). **(F)** Average percentages of PDGFRa+ cells that were also Nestin-tdT+ in *Myrf-Het;Nestin-tdT* and *Myrf-cKO;Nestin-tdT* rostral (sections 1–5) and caudal (sections 6–10) sections (*n* = 3–4, error bars = sem, Two-way ANOVA). **(G)** Representative images of *Myrf-Het;Nestin-tdT* and *Myrf-cKO;Nestin-tdT* coronal brain sections immunostained for CC1 (in green) and Nes-TdT (magenta) after 4-week recovery (Cup6w + 4w). **(H)** Quantification of the percentages of CC1+ cells that were also Nestin-tdT+ in *Myrf-Het;Nestin-tdT* and *Myrf-cKO;Nestin-tdT* coronal sections (*n* = 5, lines and shading represent mean ± sem, **p < 0.001, Two-way ANOVA). **(I)** Average percentages of CC1+ cells that were also Nestin-tdT+ in *Myrf-Het;Nestin-tdT* and *Myrf-cKO;Nestin-tdT* rostral (sections 1–5) and caudal (sections 6–10) sections (*n* = 5, error bars = sem, Two-way ANOVA).

To determine whether this enhanced NSC-progeny contribution was sustained at later times, we examined mice that had recovered for 4 weeks (Cup6w + 4w) (Figure 4 and Supplementary Figure 3). Again, qualitative assessment of remyelination by MBP and Eriochrome Cyanine stainings, and quantitative assessment of astrocyte-microglial activation based on GFAP and Iba1 immunostaining, were similar for *Myrf-Het;Nestin-tdT* and *Myrf-cKO;Nestin-tdT* mice (Figure 4A and Supplementary Figures 3A, F, G). Quantification of the number of Nestin-tdT+ cells localized in the corpus callosum still showed increased recruitment in *Myrf-cKO;Nestin-tdT* tissues compared to controls but to a lower extent (1.4-fold) (167 ± 60 cells/mm^2^ vs. 115 ± 24 cells/mm^2^, respectively, Figures 3B, C). We checked if NSC-derived cell contribution to PDGFRa+ or CC1+ pools were enhanced at this later time point (Figures 4D–I and Supplementary Figures 3B–E). At Cup6w + 4w, the percentage of Nestin-tdT+ cells that were PDGFRa+ was increased in *Myrf-cKO;Nestin-tdT* compared to *Myrf-Het;Nestin-tdT* CC sections (6.7 ± 2.7% and 3.3 ± 0.8%, respectively, Figure 4E; 52 ± 19 cells/mm^2^ vs. 25 ± 6 cells/mm^2^, respectively, Supplementary Figure 3B). These results suggest that at later time points the recruited NSC-derived cells contribute to repopulating the adult OPC pool in the corpus callosum. The increased contribution of Nestin-tdT+ to CC1+ OLs in the CC of *Myrf-cKO;Nestin-tdT* compared to controls was maintained at Cup6w + 4w (4.9 ± 2.4% vs. 2.9 ± 0.8%, respectively, Figure 4H; 52 ± 26 cells/mm^2^ vs. 30 ± 8 cells/mm^2^, respectively, Supplementary Figure 3D). These results suggest that enhanced NSC-progeny contribution is sustained, but not enhanced, at later recovery times. While these effects were significant, one caveat is that both results were principally driven by a single *Myrf-cKO;Nestin-tdT* mouse that exhibited a markedly increased recruitment of Nestin-tdT+ cells into the corpus callosum despite a normal demyelination/remyelination MBP pattern.

Koenning et al. (2012) previously showed that ablation of *Myrf* in OLs resulted in increased apoptosis in oligodendroglial cells at 4–6 week-post-tamoxifen. Because pOPC differentiation is blocked in our *Myrf-cKO;Nestin-tdT* mouse line, we checked if cells blocked in a progenitor stage also underwent apoptosis in our knock-out. We did not detect any difference in Caspase3+ and PDGFRa+ cells in *Myrf-Het;Nestin-tdT* and *Myrf-cKO;Nestin-tdT* mice at either 2 weeks (Supplementary Figure 2H) or 4 weeks (Supplementary Figure 3H) after cuprizone treatment. Either the pOPCs were maintained as progenitors in the CNS of *Myrf-cKO;Nestin-tdT* animals or our low recombination rate (29.7 ± 3.6%, Supplementary Figure 1C) did not allow us to detect subtle changes in apoptosis.

Taken together, our results indicate that NSC-derived cell recruitment to and differentiation into OLs is enhanced in the demyelinating corpus callosum when pOPC differentiation is impaired. This increased NSC-progeny contribution appeared to be more important at an early time-point, suggesting that timing is essential, allowing NSC-derived cells to be competitive against pOPCs right at the start of the repair.

## Discussion

Here, we have used dual reporter *NG2-EGFP;Nestin-tdT* mice to confirm that both pOPCs and NSCs contribute to repair of the corpus callosum following cuprizone-induced demyelination (Menn et al., 2006; Xing et al., 2014; Brousse et al., 2015; Butti et al., 2019). Similar to previous studies, we showed that NG2-EGFP+ pOPCs contribute more than Nestin-tdT+ NSC-derived cells to the pools of PDGFRa+ progenitors and CC1+ OLs during repair (Xing et al., 2014; Brousse et al., 2015; Serwanski et al., 2018; Butti et al., 2019). Due to its lower recombination efficiency, the NG2-EGFP reporter line likely significantly under-estimated the contribution of pOPCs to repair.

While cuprizone can result in variable demyelination, depending on the amount of food consumption (Toomey et al., 2021), in our studies a 6-week cuprizone treatment induced extensive and comparable demyelination in the corpus callosum of each replicate. These results strongly suggest that cuprizone-induced demyelination was similar in all mice terminated at later recovery times. Demyelination was more extensive in caudal than in rostral regions, as noted in earlier reports (Wu et al., 2008; Xie et al., 2010; Tagge et al., 2016; Zhang et al., 2019; Brousse et al., 2021; Toomey et al., 2021). In contrast to prior studies showing that the contribution of NSC-progeny to CC1+ OLs in the CC exhibited regional differences (Menn et al., 2006; Ortega et al., 2013; Xing et al., 2014; Brousse et al., 2015, 2021; Butti et al., 2019), we found an uniform contribution, from caudal to rostral sections of the CC (Figure 1J).

Taking advantage of our *Myrf-cKO;Nestin-tdT* mouse line, we then addressed if OLs derived from pOPCs and NSCs compete to remyelinate the same demyelinated regions. If remyelination by pOPCs and NSC-derived cells is restricted to distinct areas, blocking pOPC differentiation should not affect the recruitment and differentiation of NSC-derived OLs. Conversely, if pOPCs and NSC-derived cells compete for repair, preventing pOPC differentiation should allow NSC-derived cells more time to reach the demyelinated areas and differentiate into OLs. Indeed, at an early recovery time point (Cup6w + 2w), NSC-progeny recruitment and differentiation were increased twofold in knock-out *Myrf-cKO;Nestin-tdT* compared to control *Myrf-Het;Nestin-tdT* mice. This increase was also consistent from rostral to caudal regions, again suggesting no regional differences in our model. At a later recovery time point (Cup6w + 4w), this effect was maintained but not further increased. This is consistent with previous papers showing that the peak of pOPC and NSC-progeny mobilization is 2 weeks post cuprizone diet (Brousse et al., 2015).

Our results confirmed that OLs derived from pOPCs and NSCs are competing for repair in the corpus callosum. Our data complements a prior study showing ablation of NSCs had no impact on remyelination in CC, implying that pOPCs could overtake and repair regions close to the SVZ usually colonized by NSC-progeny (Butti et al., 2019). They also suggest that timing is essential: NSC-derived cells can only contribute to repair if they reach the demyelinating regions early, presumably before pOPCs, already localized on site, have fully remyelinated the lesion. Newly-myelinating pOPCs could physically block the recruitment and differentiation of NSC-derived cells, to maintain a homeostatic myelinated network (Hughes et al., 2013). They could also block the NSC contribution, directly by secreting inhibitory cues or indirectly by decreasing microglial activity once remyelination is engaged (Ribeiro Xavier et al., 2015; Sato, 2015; Vay et al., 2018). Previous reports also suggest that early recruitment of NSC-derived cells to demyelinated areas could prevent axonal damage, even without directly participating in remyelination (Butti et al., 2019). While we have only measured CC1+ OLs, it seems likely that OLs derived from the NSC pool are directly contributing to remyelination, as previously shown by Samanta et al. (2015) and Butti et al. (2019). Electron microscopy experiments would be useful to validate this point. NSC-derived cells, especially when recruited late, also likely contribute to reconstituting the pool of pOPCs, which may be depleted in demyelinated areas (Serwanski et al., 2018).

The NSC contribution to repair when pOPC differentiation was blocked was significant although limited. One likely explanation is the unexpectedly low recombination efficiency of the *NG2-CreER*™** line, which was only ∼30% in our hands vs. 45–50% in a prior report (Zhu et al., 2011). This efficiency was surprisingly low given the effectiveness of our tamoxifen treatment in activating the *Nestin-FlpoER*^*T*2^ driver. If more than half of the pOPCs indeed escaped recombination and therefore retained the ability to differentiate into remyelinating OLs, they may well have substantially constrained the efficacy of remyelination by NSC-derived cells. The modest *NG2-CreER*™** mediated recombination may also explain the lack of an evident increase in apoptosis of PDGFRa+ progenitors in the knock-out *Myrf-cKO;Nestin-tdT* mice. This contrasts with a prior report in which apoptosis was detected when pOPC differentiation was blocked more efficiently (Duncan G. J. et al., 2018). In the future, it will be of interest to determine whether the contribution of NSC-progeny in remyelination would be increased further still using a more efficient OPC-specific Cre driver line to ablate *Myrf* [e.g., *Pdgfra-CreER^(T)^*] (Kang et al., 2010).

The signals that activate NSCs and recruit their progeny into demyelinated white matter lesions are not known. We and others have shown that NSC differentiation into the oligodendroglial cell lineage is regulated in part by sonic hedgehog (Shh) signaling (Lai et al., 2003; Ahn and Joyner, 2005; Samanta et al., 2015; Sanchez and Armstrong, 2018; Namchaiw et al., 2019; Delgado et al., 2021). In demyelinating models, the effects of Shh signaling on remyelination are complex. Inhibition of the Shh induced transcription factor *Gli1* increases NSC recruitment and remyelination (Samanta et al., 2015; Namchaiw et al., 2019), whereas global Shh upregulation can both prevent disease progression and enhance pOPC remyelinating capacities (Ferent et al., 2013; Xiao et al., 2021). In addition to its role in the developmental and postnatal oligodendroglial cell lineage (Ortega et al., 2012; Sanchez and Armstrong, 2018), Shh can further regulate pOPC population in adult tissues, suggesting a larger role of Shh in the oligodendroglial lineage and during remyelination. Indeed, Shh activation can induce pOPC proliferation, migration and early differentiation in the adult mouse brain (Loulier et al., 2006; Merchán et al., 2007). Recent studies also suggest that microglia influence the proliferation and differentiation of NSCs (Ribeiro Xavier et al., 2015; Sato, 2015; Vay et al., 2018). Microgliosis is an essential component of the cuprizone model, driving the initial demyelination, participating in myelin debris phagocytosis and organizing remyelination (Marzan et al., 2021; Sen et al., 2022). By being among the first cells within demyelinating lesions, microglia may serve to activate NSCs and/or recruit NSC-derived cells directly or indirectly. Recently, it has been proposed that NSCs could also have an immunomodulatory role, favoring a neuroprotective microglial profile and limiting their pro-inflammatory profile (Brousse et al., 2021).

Reactive pOPCs, transitioning into remyelinating OLs, are also candidates to secrete signals that recruit NSCs into demyelinating lesions. Indeed, during cuprizone-induced demyelinating treatment, adult OPCs secrete many cytokines and growth factors that could activate and recruit NSC-derived cells (Gadani et al., 2015; Moyon et al., 2015; Madsen et al., 2020). In this model, blockade of pOPC differentiation in the knock-out *Myrf-cKO;Nestin-tdT* mice might block release of such signals and account for the limited activation of NSCs and recruitment of NSC-progeny.

In conclusion, our results show that pOPCs compete with NSC-derived cells for remyelination of the corpus callosum. Our work suggests that with aging and disease, when pOPC differentiation is limited, the contribution of NSC-derived OLs may increase–even at sites far from the SVZ, given the migratory abilitie of NSCs and their progeny (Ahlenius et al., 2009; Kalamakis et al., 2019). Similarly, in diseases such as Multiple Sclerosis, in which pOPCs can be depleted in demyelinated lesions (Boyd et al., 2013; Heß et al., 2020), targeting the NSC contribution to repair may be a promising therapeutic strategy.

## Data availability statement

The original contributions presented in this study are included in this article/Supplementary material, further inquiries can be directed to the corresponding authors.

## Ethics statement

The animal study was reviewed and approved by Institutional Animal Care and Use Committee (IACUC) of New York University (NYU) Langone Health.

## Author contributions

SM and MH: data acquisition. SM: data analysis, visualization, and original draft. SM and JS: conceptualization, writing, and editing. JS: funding acquisition and supervision. All authors contributed to the article and approved the submitted version.

## Supplementary material

Supplementary Figure 1 Generation and validation of an inducible *NG2-EGFP;Nestin-tdT* dual reporter line to assess the contributions of neural stem cell progeny and parenchymal oligodendrocyte progenitor cells (pOPCs) to remyelination. **(A)** Representative images of *NG2-EGFP;Nestin-tdT* coronal brain section, 1-week post-oil or post-tamoxifen induction. **(B)** Representative images of *NG2-EGFP;Nestin-tdT* coronal brain section, 1-week post-tamoxifen induction, immunostained for Nestin (in white) and Nes-TdT (in magenta). Quantification of the percentage of Nestin-tdT + &Nestin + cells/Nestin + cells after oil or tamoxifen induction in *NG2-EGFP;Nestin-tdT* mice (*n* = 3, error bars = sem). **(C)** Representative confocal images of *NG2-EGFP;Nestin-tdT* coronal brain section, 1-week post-tamoxifen induction, immunostained for NG2 (in white) and NG2-EGFP (in green). Quantification of the percentage of NG2-EGFP + &NG2 + cells/NG2+ cells after oil or tamoxifen induction in *NG2-EGFP;Nestin-tdT* mice (*n* = 3, error bars = sem). **(D)** Quantification of the densities of PDGFRa+ cells that were also NG2-EGFP+ or Nestin-tdT+ for all rostral to caudal CC sections (*n* = 4, lines and shading represent mean ± sem, ****p* < 0.001, Two-way ANOVA). **(E)** Average densities of PDGFRa+ cells that were also NG2-EGFP+ or Nestin-tdT+ for rostral (sections 1–5) and caudal (sections 6–10) sections (*n* = 4, error bars = sem, ****p* < 0.001, ***p* < 0.01, Student’s *t*-test). **(F)** Quantification of the densities of CC1+ cells that were also NG2-EGFP+ or Nestin-tdT+ for all rostral to caudal CC sections (*n* = 4, lines and shading represent mean ± sem, ****p* < 0.001, Two-way ANOVA). **(G)** Average densities of CC1+ cells that were also NG2-EGFP+ or Nestin-tdT+ for rostral (sections 1–5) and caudal (sections 6–10) sections (*n* = 4, error bars = sem, **p* < 0.05, Student’s *t*-test).

Supplementary Figure 2 The contribution of neural stem cell-derived oligodendrocytes (OLs) to remyelination increases at 2 weeks of recovery when parenchymal oligodendrocyte progenitor cell differentiation is blocked. **(A)** Representative images of *Myrf-Het;Nestin-tdT* and *Myrf-cKO;Nestin-tdT* coronal brain sections stained with Eriochrome Cyanine (Erio. Cya., in blue) after 2-week recovery (Cup6w + 2w) (sections 2 and sections 9 of the same animals). **(B)** Quantification of the densities of PDGFRa+ cells that were also Nestin-tdT+ in *Myrf-Het;Nestin-tdT* and *Myrf-cKO;Nestin-tdT* coronal sections (*n* = 3–4, lines and shading represent mean ± sem, Two-way ANOVA). **(C)** Average densities of PDGFRa+ cells that were also Nestin-tdT+ in *Myrf-Het;Nestin-tdT* and *Myrf-cKO;Nestin-tdT* rostral (sections 1–5) and caudal (sections 6–10) sections (*n* = 3–4, error bars = sem, Two-way ANOVA). **(D)** Quantification of the densities of CC1+ cells that were also Nestin-tdT+ in *Myrf-Het;Nestin-tdT* and *Myrf-cKO;Nestin-tdT* coronal sections (*n* = 6–7, lines and shading represent mean ± sem, ****p* < 0.001, Two-way ANOVA). **(E)** Average densities of CC1+ cells that were also Nestin-tdT+ in *Myrf-Het;Nestin-tdT* and *Myrf-cKO;Nestin-tdT* rostral (sections 1–5) and caudal (sections 6–10) sections (*n* = 6–7, error bars = sem, **p* < 0.05, Two-way ANOVA). **(F)** Quantification of the GFAP+ intensity in the CC of *Myrf-Het;Nestin-tdT* and *Myrf-cKO;Nestin-tdT* brain sections (*n* = 5, error bars = sem, Student’s *t*-test). **(G)** Quantification of the Iba1+ intensity in the corpus callosum of *Myrf-Het;Nestin-tdT* and *Myrf-cKO;Nestin-tdT* brain sections (*n* = 5, error bars = sem, Student’s *t*-test). **(H)** Quantification of the percentage of PDGFRa+ and Caspase3+ cells in the corpus callosum of *Myrf-Het;Nestin-tdT* and *Myrf-cKO;Nestin-tdT* brain sections (*n* = 3–4, error bars = sem, Student’s *t*-test).

Supplementary Figure 3 The contribution of neural stem cell-derived oligodendrocytes (OLs) to remyelination remains elevated at 4 weeks of recovery when parenchymal oligodendrocyte progenitor cell differentiation is blocked. **(A)** Representative images of *Myrf-Het;Nestin-tdT* and *Myrf-cKO;Nestin-tdT* coronal brain sections stained with Eriochrome Cyanine (Erio. Cya., in blue) after 4-week recovery (Cup6w + 4w) (sections 2 and sections 9 of the same animals). **(B)** Quantification of the densities of PDGFRa+ cells that were also Nestin-tdT+ in *Myrf-Het;Nestin-tdT* and *Myrf-cKO;Nestin-tdT* coronal sections (*n* = 3–4, lines and shading represent mean ± sem, ****p* < 0.001, Two-way ANOVA). **(C)** Average densities of PDGFRa+ cells that were also Nestin-tdT+ in *Myrf-Het;Nestin-tdT* and *Myrf-cKO;Nestin-tdT* rostral (sections 1–5) and caudal (sections 6–10) sections (*n* = 3–4, error bars = sem, Two-way ANOVA). **(D)** Quantification of the densities of CC1+ cells that were also Nestin-tdT+ in *Myrf-Het;Nestin-tdT* and *Myrf-cKO;Nestin-tdT* coronal sections (*n* = 5, lines and shading represent mean ± sem, ***p* < 0.01, Two-way ANOVA). **(E)** Average densities of CC1+ cells that were also Nestin-tdT+ in *Myrf-Het;Nestin-tdT* and *Myrf-cKO;Nestin-tdT* rostral (sections 1–5) and caudal (sections 6–10) sections (*n* = 5, error bars = sem, Two-way ANOVA). **(F)** Quantification of the GFAP+ intensity in the CC of *Myrf-Het;Nestin-tdT* and *Myrf-cKO;Nestin-tdT* brain sections (*n* = 3–4, error bars = sem, Student’s *t*-test). **(G)** Quantification of the Iba1+ intensity in the CC of *Myrf-Het;Nestin-tdT* and *Myrf-cKO;Nestin-tdT* brain sections (*n* = 3–4, error bars = sem, Student’s *t*-test). **(H)** Quantification of the percentage of PDGFRa+ and Caspase3+ cells in the corpus callosum of *Myrf-Het;Nestin-tdT* and *Myrf-cKO;Nestin-tdT* brain sections (*n* = 3–4, error bars = sem, Student’s *t*-test).
