# Supplementary figures and images for: Neural stem cells and oligodendrocyte progenitor cells compete for remyelination in the corpus callosum
Source: Front Cell Neurosci. 2023 Jan 26;17:1114781. doi: 10.3389/fncel.2023.1114781 (PMC9909070; doi:10.3389/fncel.2023.1114781)

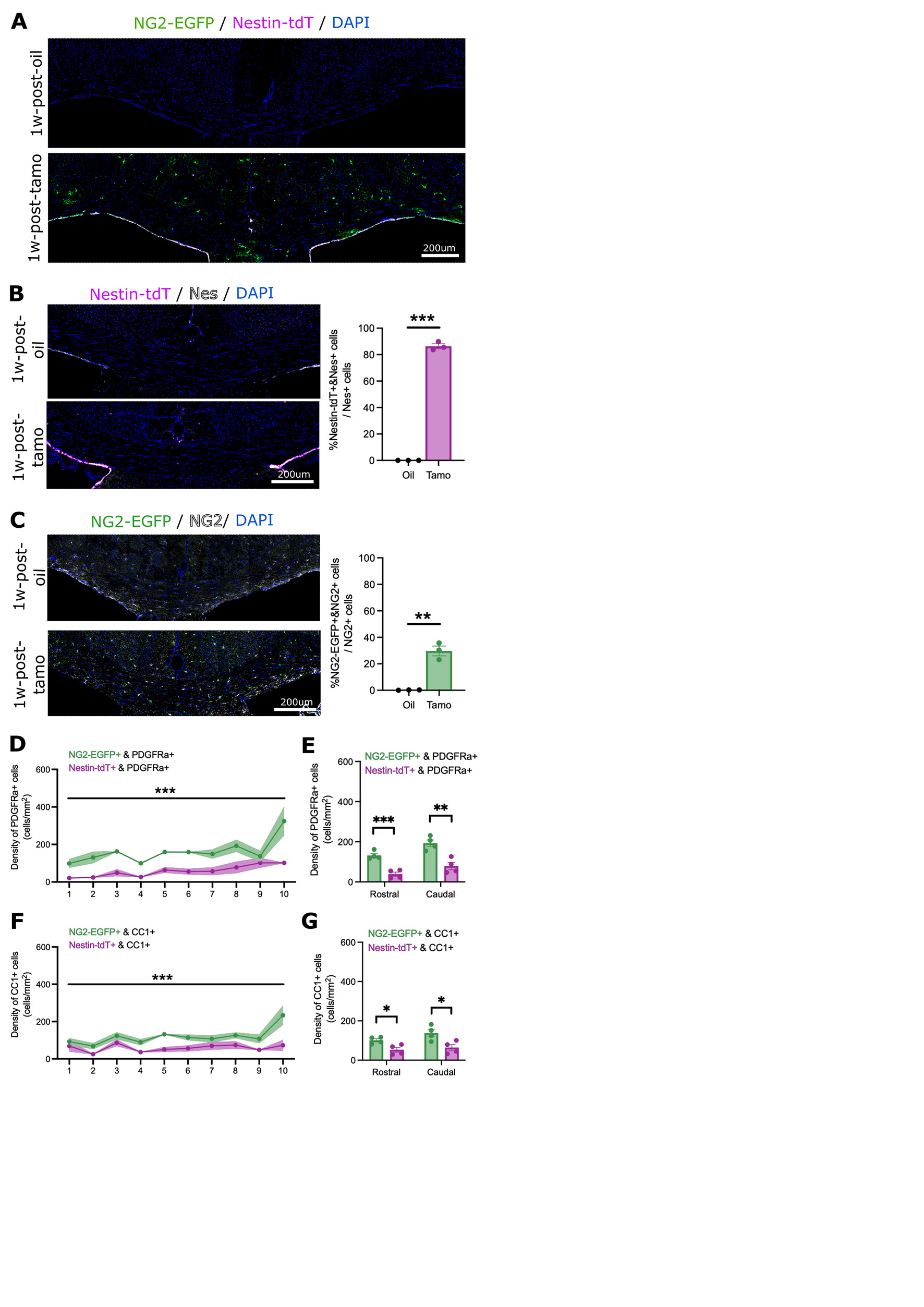

Supplement: Supplementary Figure 1 — Generation and validation of an inducible NG2-EGFP;Nestin-tdT dual reporter line to assess the contributions of neural stem cell progeny and parenchymal oligodendrocyte progenitor cells (pOPCs) to remyelination. (A) Representative images of NG2-EGFP;Nestin-tdT coronal brain section, 1-week post-oil or post-tamoxifen induction. (B) Representative images of NG2-EGFP;Nestin-tdT coronal brain section, 1-week post-tamoxifen induction, immunostained for Nestin (in white) and Nes-TdT (in magenta). Quantification of the percentage of Nestin-tdT + &Nestin + cells/Nestin + cells after oil or tamoxifen induction in NG2-EGFP;Nestin-tdT mice (n = 3, error bars = sem). (C) Representative confocal images of NG2-EGFP;Nestin-tdT coronal brain section, 1-week post-tamoxifen induction, immunostained for NG2 (in white) and NG2-EGFP (in green). Quantification of the percentage of NG2-EGFP + &NG2 + cells/NG2+ cells after oil or tamoxifen induction in NG2-EGFP;Nestin-tdT mice (n = 3, error bars = sem). (D) Quantification of the densities of PDGFRa+ cells that were also NG2-EGFP+ or Nestin-tdT+ for all rostral to caudal CC sections (n = 4, lines and shading represent mean ± sem, ***p < 0.001, Two-way ANOVA). (E) Average densities of PDGFRa+ cells that were also NG2-EGFP+ or Nestin-tdT+ for rostral (sections 1–5) and caudal (sections 6–10) sections (n = 4, error bars = sem, ***p < 0.001, **p < 0.01, Student’s t-test). (F) Quantification of the densities of CC1+ cells that were also NG2-EGFP+ or Nestin-tdT+ for all rostral to caudal CC sections (n = 4, lines and shading represent mean ± sem, ***p < 0.001, Two-way ANOVA). (G) Average densities of CC1+ cells that were also NG2-EGFP+ or Nestin-tdT+ for rostral (sections 1–5) and caudal (sections 6–10) sections (n = 4, error bars = sem, *p < 0.05, Student’s t-test). [file Image_1.JPEG]

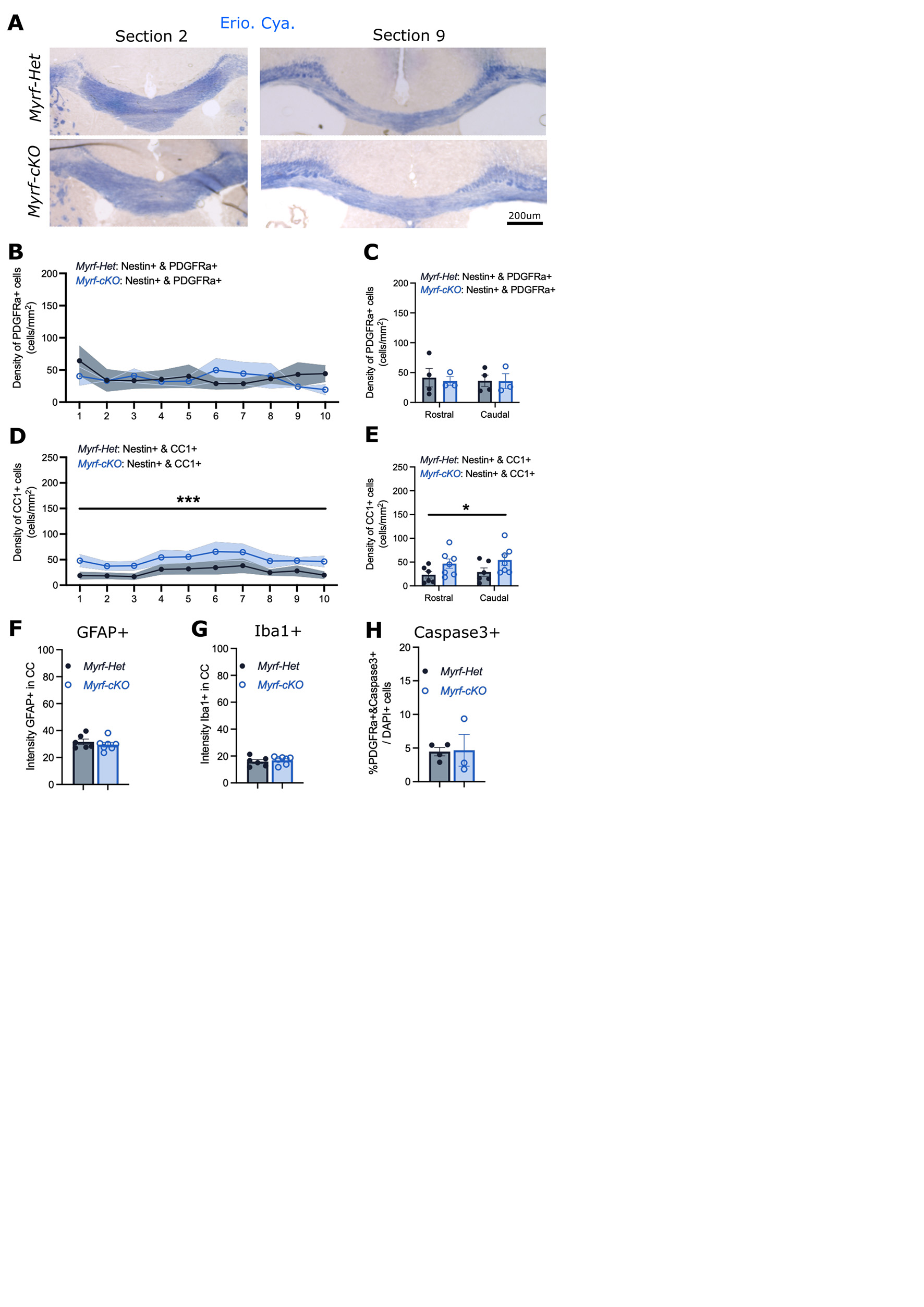

Supplement: Supplementary Figure 2 — The contribution of neural stem cell-derived oligodendrocytes (OLs) to remyelination increases at 2 weeks of recovery when parenchymal oligodendrocyte progenitor cell differentiation is blocked. (A) Representative images of Myrf-Het;Nestin-tdT and Myrf-cKO;Nestin-tdT coronal brain sections stained with Eriochrome Cyanine (Erio. Cya., in blue) after 2-week recovery (Cup6w + 2w) (sections 2 and sections 9 of the same animals). (B) Quantification of the densities of PDGFRa+ cells that were also Nestin-tdT+ in Myrf-Het;Nestin-tdT and Myrf-cKO;Nestin-tdT coronal sections (n = 3–4, lines and shading represent mean ± sem, Two-way ANOVA). (C) Average densities of PDGFRa+ cells that were also Nestin-tdT+ in Myrf-Het;Nestin-tdT and Myrf-cKO;Nestin-tdT rostral (sections 1–5) and caudal (sections 6–10) sections (n = 3–4, error bars = sem, Two-way ANOVA). (D) Quantification of the densities of CC1+ cells that were also Nestin-tdT+ in Myrf-Het;Nestin-tdT and Myrf-cKO;Nestin-tdT coronal sections (n = 6–7, lines and shading represent mean ± sem, ***p < 0.001, Two-way ANOVA). (E) Average densities of CC1+ cells that were also Nestin-tdT+ in Myrf-Het;Nestin-tdT and Myrf-cKO;Nestin-tdT rostral (sections 1–5) and caudal (sections 6–10) sections (n = 6–7, error bars = sem, *p < 0.05, Two-way ANOVA). (F) Quantification of the GFAP+ intensity in the CC of Myrf-Het;Nestin-tdT and Myrf-cKO;Nestin-tdT brain sections (n = 5, error bars = sem, Student’s t-test). (G) Quantification of the Iba1+ intensity in the corpus callosum of Myrf-Het;Nestin-tdT and Myrf-cKO;Nestin-tdT brain sections (n = 5, error bars = sem, Student’s t-test). (H) Quantification of the percentage of PDGFRa+ and Caspase3+ cells in the corpus callosum of Myrf-Het;Nestin-tdT and Myrf-cKO;Nestin-tdT brain sections (n = 3–4, error bars = sem, Student’s t-test). [file Image_2.JPEG]

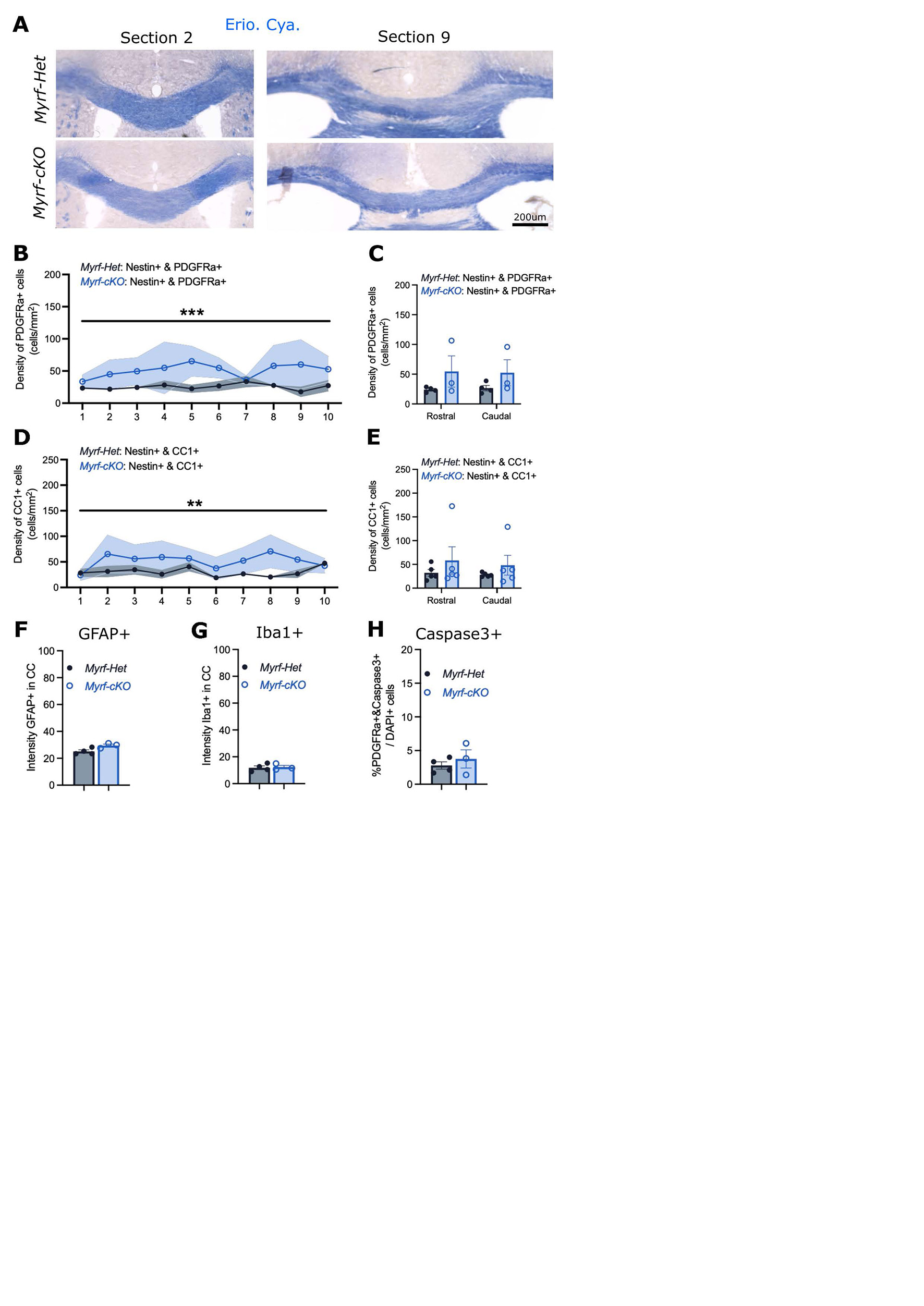

Supplement: Supplementary Figure 3 — The contribution of neural stem cell-derived oligodendrocytes (OLs) to remyelination remains elevated at 4 weeks of recovery when parenchymal oligodendrocyte progenitor cell differentiation is blocked. (A) Representative images of Myrf-Het;Nestin-tdT and Myrf-cKO;Nestin-tdT coronal brain sections stained with Eriochrome Cyanine (Erio. Cya., in blue) after 4-week recovery (Cup6w + 4w) (sections 2 and sections 9 of the same animals). (B) Quantification of the densities of PDGFRa+ cells that were also Nestin-tdT+ in Myrf-Het;Nestin-tdT and Myrf-cKO;Nestin-tdT coronal sections (n = 3–4, lines and shading represent mean ± sem, ***p < 0.001, Two-way ANOVA). (C) Average densities of PDGFRa+ cells that were also Nestin-tdT+ in Myrf-Het;Nestin-tdT and Myrf-cKO;Nestin-tdT rostral (sections 1–5) and caudal (sections 6–10) sections (n = 3–4, error bars = sem, Two-way ANOVA). (D) Quantification of the densities of CC1+ cells that were also Nestin-tdT+ in Myrf-Het;Nestin-tdT and Myrf-cKO;Nestin-tdT coronal sections (n = 5, lines and shading represent mean ± sem, **p < 0.01, Two-way ANOVA). (E) Average densities of CC1+ cells that were also Nestin-tdT+ in Myrf-Het;Nestin-tdT and Myrf-cKO;Nestin-tdT rostral (sections 1–5) and caudal (sections 6–10) sections (n = 5, error bars = sem, Two-way ANOVA). (F) Quantification of the GFAP+ intensity in the CC of Myrf-Het;Nestin-tdT and Myrf-cKO;Nestin-tdT brain sections (n = 3–4, error bars = sem, Student’s t-test). (G) Quantification of the Iba1+ intensity in the CC of Myrf-Het;Nestin-tdT and Myrf-cKO;Nestin-tdT brain sections (n = 3–4, error bars = sem, Student’s t-test). (H) Quantification of the percentage of PDGFRa+ and Caspase3+ cells in the corpus callosum of Myrf-Het;Nestin-tdT and Myrf-cKO;Nestin-tdT brain sections (n = 3–4, error bars = sem, Student’s t-test). [file Image_3.jpg]
